# Computed Tomography-Based Radiomics for Differentiation of Thymic Epithelial Tumors and Lymphomas in Anterior Mediastinum

**DOI:** 10.3389/fonc.2022.869982

**Published:** 2022-05-13

**Authors:** Wenzhang He, Chunchao Xia, Xiaoyi Chen, Jianqun Yu, Jing Liu, Huaxia Pu, Xue Li, Shengmei Liu, Xinyue Chen, Liqing Peng

**Affiliations:** ^1^ Department of Radiology, West China Hospital, Sichuan University, Chengdu, China; ^2^ Computed Tomography (CT) Collaboration, Siemens Healthineers, Chengdu, China

**Keywords:** radiomics, thymic epithelial tumors, lymphoma, anterior mediastinum, computed tomography

## Abstract

**Objective:**

To investigate the differential diagnostic performance of computed tomography (CT)-based radiomics in thymic epithelial tumors (TETs) and lymphomas in anterior mediastinum.

**Methods:**

There were 149 patients with TETs and 93 patients with lymphomas enrolled. These patients were assigned to a training set (n = 171) and an external validation set (n = 71). Dedicated radiomics prototype software was used to segment lesions on preoperative chest enhanced CT images and extract features. The multivariable logistic regression algorithm was used to construct three models according to clinico-radiologic features, radiomics features, and combined features, respectively. Performance of the three models was compared by using the area under the receiver operating characteristic curves (AUCs). Decision curve analysis was used to evaluate clinical utility of the three models.

**Results:**

For clinico-radiologic model, radiomics signature model, and combined model, the AUCs were 0.860, 0.965, 0.975 and 0.843, 0.961, 0.955 in the training cohort and the test cohort, respectively (all *P*<0.05). The accuracies of each model were 0.836, 0.895, 0.918 and 0.845, 0.901, 0.859 in the two cohorts, respectively (all *P*<0.05). Compared with the clinico-radiologic model, better diagnostic performances were found in the radiomics signature model and the combined model.

**Conclusions:**

Radiomics signature model and combined model exhibit outstanding and comparable differential diagnostic performances between TETs and lymphomas. The CT-based radiomics analysis might serve as an effective tool for accurately differentiating TETs from lymphomas before treatment.

## Introduction

Thymic epithelial tumors (TETs), including thymomas and thymic carcinomas, are derived from thymic epithelial cells and are relatively rare mediastinal tumors (1). Lymphoma is divided into Hodgkin’s lymphoma and non-Hodgkin’s lymphoma (2). It is a serious malignant tumor derived from the lymphatic system, accounting for 3.2% of newly diagnosed neoplasms and 2.9% of cancer-specific mortality in China in 2018 (3). The difference is that anterior mediastinum is not a common primary site of lymphoma, but a common site of TETs (4).

For TETs, even the well-differentiated thymoma subtypes are considered to be malignant tumors with indolent growth, they also show potential for local invasion, pleural dissemination, and even distant metastasis. Therefore, once TETs are detected, surgical treatment is the mainstay treatment, and radiotherapy, chemotherapy, and other adjuvant treatments are usually supplements depending on final pathology and clinical stage according to relating treatment guidelines (5, 6). On the contrary, radiotherapy and chemotherapy are the first-line options for the treatment of lymphoma while surgical treatment is not recommended according to treatment guidelines for malignant lymphoma in 2021 in China (2, 7).

TETs and lymphomas in anterior mediastinum usually manifest as soft-tissue masses or nodules with similar imaging features (8). In clinical practice, based on typical clinical manifestations and traditional imaging findings, such as with or without myasthenia gravis, age of onset, lymphadenopathy, and imaging features, partial patients could be accurately diagnosed (9). However, the preoperative accurate diagnosis of the two types of neoplasms is usually influenced by the overlap of clinical and radiologic manifestations, the heterogeneity of disease manifestations and difficulty to obtain pathological tissues due to the complexity of the anterior mediastinum. Based on previous computed tomography (CT)-based routine preoperative examinations, although preoperative biopsy is recommended, non-essential or non-therapeutic thymectomy is not uncommon for patients with anterior mediastinal space-occupying lesions (4, 6).

Therefore, it is of great value to distinguish TETs and lymphomas non-invasively before management. Recently, radiomics with machine learning algorithms to mine high-dimensional invisible and interpretable image information into objective and quantitative mathematical data has been an established tool in the differential diagnosis of nodules/masses, prediction of tumor pathological subtypes, evaluation of disease sensitivity to treatment (10–14). According to the literature, there were only a few radiomics-based studies on anterior mediastinal diseases, especially TETs (11, 15). Some previous studies have verified that radiomics could be used for the grading of TETs and the diagnosis of partial anterior mediastinal nodules or masses (11, 15, 16). CT is commonly used in the diagnosis of chest diseases. Compared with traditional CT examination and clinical data, radiomics features extracted from CT images could provide more diagnostic information. Therefore, patients of lymphoma with atypical clinical and radiologic manifestations may avoid unnecessary surgery and receive effective treatment earlier. 

Thus, we aimed to investigate the differential diagnostic performance of CT-based radiomics in thymic epithelial tumors (TETs) and lymphomas in anterior mediastinum.

## Materials and Methods

### Subjects

The ethics committee of the participating center approved the study. The need for informed patient consent was waived because of the retrospective nature of the analysis and the use of anonymized data.

An author collected hospitalized patients diagnosed with TETs (including thymoma, thymic carcinoma) and lymphoma within the time range of October 2008 to March 2021 from the information management department of West China Hospital. A total of 852 patients were found, of which 332 had TETs and 520 had lymphomas. The author queried the patient’s pathological data from the Electronic Medical Records and searched for patients’ chest enhanced computed tomography (CECT) images in DICOM format in the Picture Archiving and Communication System, and the time of CT examination was within 30 days before the patient’s pathological samples were obtained. Pathological results came from thoracentesis, lymphadenectomy, or postoperative tissue. Then pathologically confirmed patients with CECT images had 195 with lymphomas and 222 with TETs.

**Figure 1:**
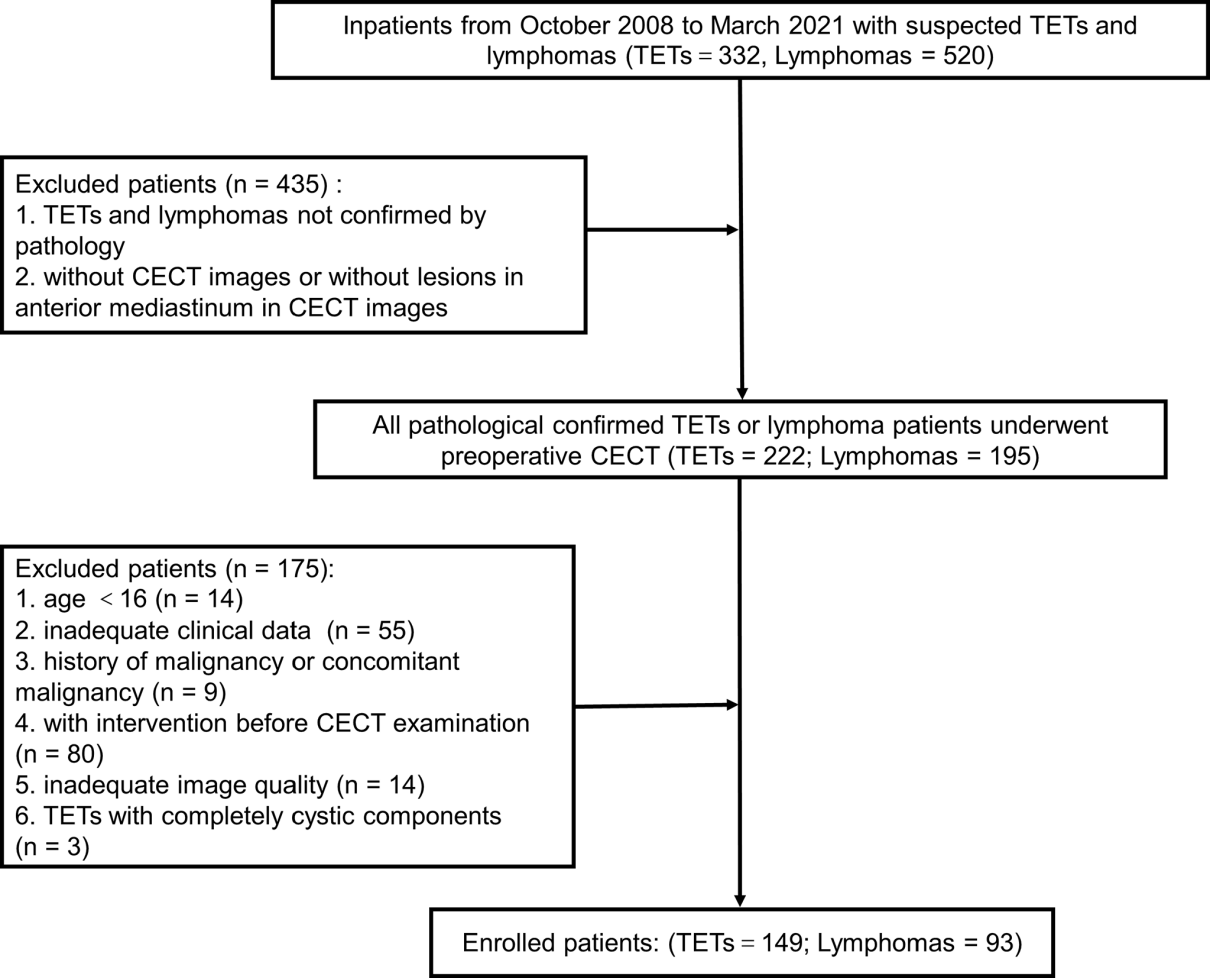
The patient selection workflow. TETs, thymic epithelial tumors; CECT, chest enhanced computed tomography.

**Figure 2:**
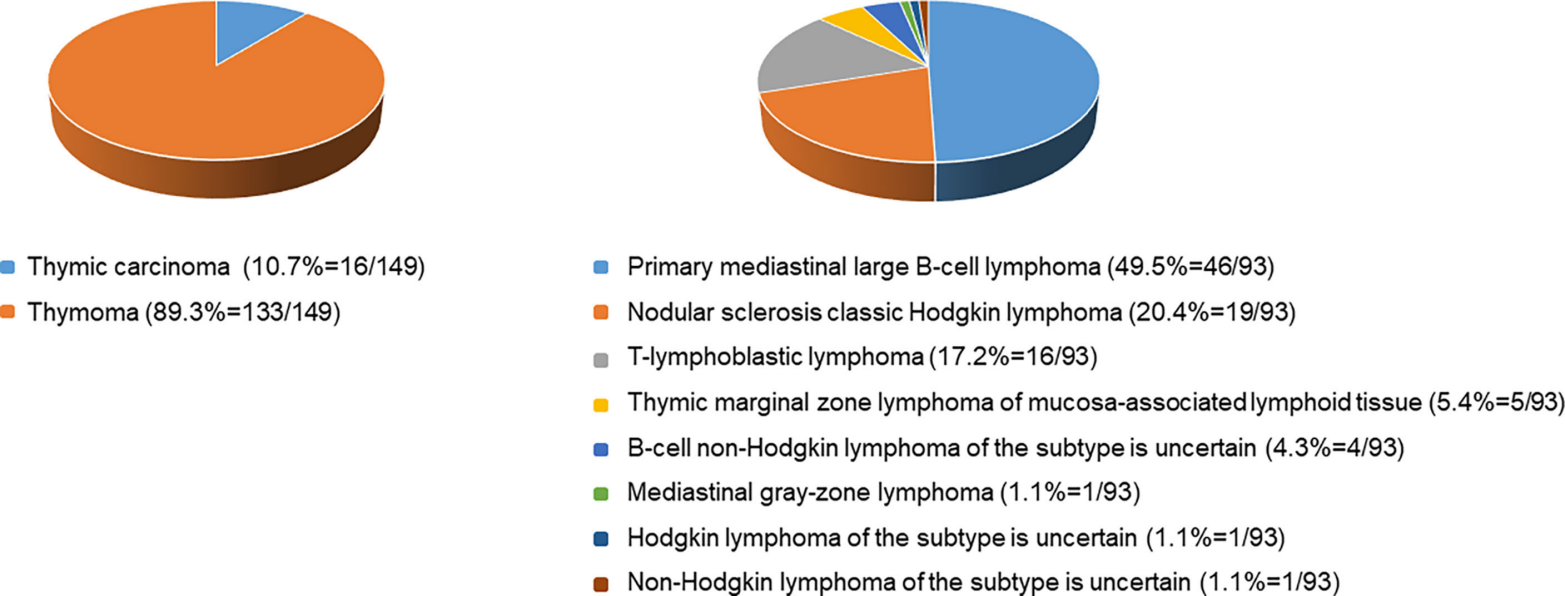
Subtype distribution of thymic epithelial tumors and lymphomas.

Each enrolled patient met the following exclusion criteria. The exclusion criteria included : 1) age<16 years old, 2) with intervention before CECT examination, 3) history of malignancy or concomitant malignancy, 4) inadequate clinical data, 5) TETs with completely cystic components, and 6) inadequate image quality. Consequently, 242 patients, including 149 patients with TETs and 93 patients with lymphomas, consisted of study cohorts. The patient selection workflow is shown in **Figure 1**. Subtype distribution of two tumors is shown in **Figure 2**.

### Clinico-Radiologic Characteristics

For TETs patients and lymphoma patients, the characteristic of P<0.10 difference between the two groups would be further used for univariate logistic regression analysis. The univariate analysis results are shown in **Tables 1** and **2**. **Table 2** showed the clinical characteristics, including clinical manifestations, symptoms, and laboratory examination results. Clinical manifestations and symptoms were collected from the first hospitalization record in the Hospital Information System by one author. Myasthenia gravis and autoimmune diseases were counted separately. In addition, autoimmune diseases including myasthenia gravis, systemic lupus erythematosus, ankylosing spondylitis, and so on, were related to thymoma (17). The laboratory results came from once test obtained before the treatment and within 3 days of the CT examination.

**Table 1:** Estimated risk of radiologic characteristics by univariate logistic regression analysis.

Variables	TETs	Lymphomas	Estimated risk	*P* valve
Max diameter (cm)	5.2 ± 2.4	9.7 ± 3.0	1.87(1.61-2.22)	<0.001
Location			
0, central	130 (87.2)	76 (81.7)	1.53(0.74-	0.200
1, peripheral	19 (12.8)	17 (18.3)	3.13)	
Morphology			
0, regular	48 (32.2)	12 (12.9)	3.21(1.64-	0.001
1, irregular	101 (67.8)	81 (87.1)	6.69)	
Fat gap			
0, present	43 (28.9)	7 (7.5)	4.98(2.26-	<0.001
1, absent	106 (71.1)	86 (92.5)	12.60)	
Small vessel			
0, absent	93 (62.4)	8 (8.6)	17.6 (8.38-	<0.001
1, present	56 (37.6)	85 (91.4)	42.00)	
Pericardial effusion			
0, absent	138 (92.6)	31 (33.3)	25.1 (12.30-	<0.001
1, present	11 (7.4)	62 (66.7)	55.60)	
Pleural effusion			
0, absent	135 (90.6)	53 (57.0)	7.28 (3.74-	<0.001
1, present	14 (9.4)	40 (43.0)	14.90)	
Necrosis			
0, absent	91 (61.1)	23 (24.7)	4.78 (2.72-	<0.001
1, present	58 (38.9)	70 (75.3)	8.62)	
Density uniformity			
0, uniform	56 (37.6)	16 (17.2)	2.90 (1.57-	<0.001
1, nonuniform	93 (62.4)	77 (82.8)	5.59)	
Boundary clarity			
0, clear	114 (76.5)	19 (20.4)	12.7 (6.88-	<0.001
1, vague	35 (23.5)	74 (79.6)	24.40)	
CT value (HU)	45.6 ± 11.4	42.2 ± 9.3	0.97 (0.94-0.99)	0.019
NEV	0.119 ± 0.079	0.088 ± 0.055	0.00 (0.00-0.05)	0.001

Max diameter, The longest diameter in the largest cross-section of the tumor; Location, Peripheral is defined as more than 2/3 of the tumor volume is located on one side of the mid-sternal line; Morphology, Lesions with round, oval, or rectangular shape are defined as regular morphology; Fat gap, Fat gaps between the nodules/masses and the ascending aorta or main pulmonary artery; Small vessel, Continuous blood pool enhancement on the chest enhanced CT image; Pericardial effusion (Pleural effusion), CT images show pericardial (pleural) thickening, pericardial (pleural) effusion, or both; Boundary clarity, Existing fuzzy boundary between the tumor and the surrounding structures, which is defined as unclear boundary; NEV, Normalized enhancement value.

**Table 2:** Estimated risk of clinical characteristics by univariate logistic regression analysis.

Variables	TETs	Lymphomas	Estimated risk	*P* valve
Age (years)	50.2 ± 12.4	31.2 ± 10.0	0.88 (0.85-0.91)	<0.001
Sex			
0, male	73 ± 49.0	42 ± 45.2	1.17 (0.69-1.97)	0.600
1, female	76 ± 51.0	51 ± 54.8		
Chest pain			
0, absent	121 (81.2)	60 (64.5)	2.38 (1.32-4.32)	0.004
1, present	28 (18.8)	33 (35.5)		
Respiratory symptom			
0, absent	105 (70.5)	31 (33.3)	5.10 (2.93-9.07)	<0.001
1, present	44 (29.5)	62 (66.7)		
B symptom			
0, absent	132 (88.6)	77 (82.8)	1.61(0.77-3.39)	0.200
1, present	17 (11.4)	16 (17.2)		
Lymphadenopathy			
0, absent	148 (99.3)	76 (81.7)	33.1(6.61-602.00)	<0.001
1, present	1 (0.7)	17 (18.3)		
Myasthenia gravis			
0, absent	110 (73.8)	92 (98.9)	0.03 (0.00-0.15)	<0.001
1, present	39 (26.2)	1 (1.1)		
Autoimmune disease			
0, absent	106 (71.1)	91 (97.8)	0.05 (0.01-0.18)	<0.001
1, present	43 (28.9)	2 (2.2)		
Red blood cell count (×10^12^/L)	4.6 ± 0.7	4.6 ± 0.6	0.93 (0.61-1.42)	0.700
Leukocyte count (×10^9^/L)	6.5 ± 2.6	14.9 ± 43.7	1.00 (0.99-1.00)	0.056
Lymphocyte count (×10^9^/L)	1.8 ± 0.7	1.3 ± 1.1	0.39 (0.25-0.59)	<0.001
Platelet count (×10^9^/L)	187.8 ± 70.0	290.0 ± 123.3	1.01 (1.01-1.02)	<0.001
Lactate dehydrogenase (IU/L)	163.5 ± 40.2	437.0 ± 385.2	1.02 (1.02-1.03)	<0.001

B symptoms is defined as the patient manifests at least one of the following three symptoms: 1) unexplained fever ＞38℃, 2) night sweats , 3) weight loss more than 10% within 6 months respiratory symptom including cough, wheezing, expectoration, chest tightness, and hemoptysis; lymphadenopathy, lymphadenopathy at physical examination.

In PACS, two radiologists (with 5 years and 10 years of experience with chest CT, respectively) worked together to analyze radiological characteristics, including morphological related features and some quantifiable features, with the naked eye in the Picture Archiving and Communication System. When there was an inconsistency, the two radiologists would reach an agreement through discussion. Normalized enhancement value (NEV) was calculated as NEV = EV*lesion*/EV*aorta*, where EV*lesion* and EV*aorta* are the CT value difference before and after enhancement in the largest cross-section of the lesion and in the lumen of the ascending aorta at the same cross-section. The CT images used to extract radiomics features and to analysis by the naked eye came from the same CT examination. When more than once CT examination was available for radiological analysis, we selected the last CT examination before surgery or biopsy.

### Chest Enhanced CT Scan

Before CECT images collection, a total of 80–120 mL (1.5 mL/kg) of iodinated contrast agent was injected *via* the antecubital vein at a flow rate of 4 mL/s. CECT scans were obtained at 40 sec after injection of contrast media. All the CECT images were acquired by standard institutional procedure protocols and stored in DICOM format. Related parameters are shown in **Table 3**. In all patients, CT images were acquired in the supine position at full inspiration.

**Table 3:** Computed tomography image acquisition parameters.

Manufacturers	Image extent (pixels)	Voxel space (mm)	Slice thickness (mm)	Voltage (kV)	Tube current (mA)
SIEMENS, n=144 PHILIPS, n=65 UIH, n=12 GE, n=21	512×512	Mean ± SD 0.707 ± 0.077 Median 0.702 Range 0.539-0.973	0.7, n= 2; 1.0, n=2 2.0, n=1; 2.5, n=1 5.0, n=220; 7.0, n=5 7.5, n=1; 8.0, n=9	80, n= 2 100, n= 70 120, n=168 140, n=2	Mean ± SD 278.6 ± 100.8 Median 267 Range 79-649

### ROI Acquisition and Radiomics Feature Extraction

**Figure 3:**
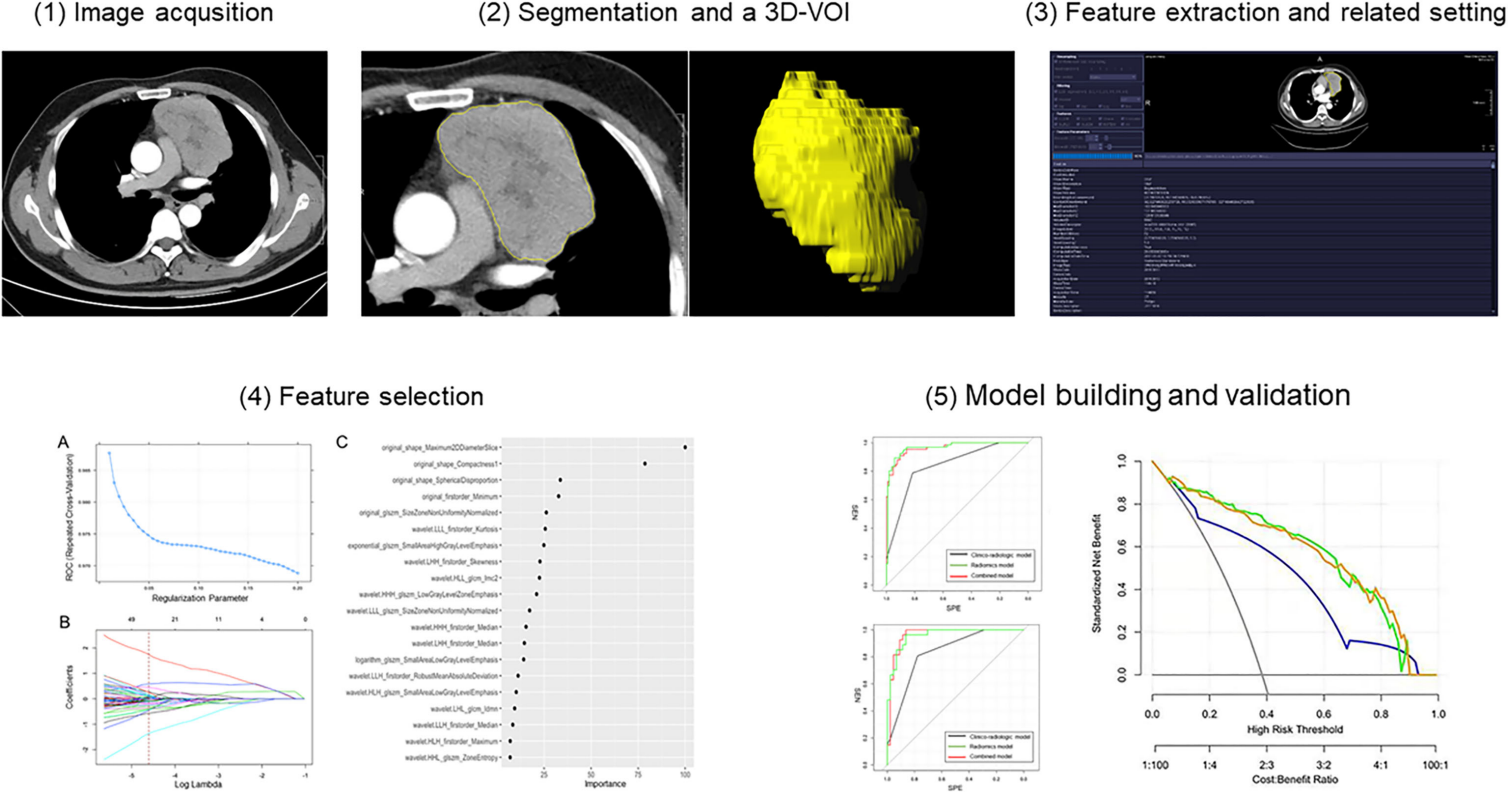
Workflow diagram of radiomics analysis. 3D-VOI, 3-dimensional volume of interest.

The CECT images in DICOM format from selected patients were segmented by using “Radiomics” (Syngo. *via* Frontier, Vision 1.0.0, Siemens, Germany), a dedicated prototype software, and this program employs an embedded 3D-printing technique in a semi-automatic manner to label the preoperative soft tissue. The overall procedures of this analysis scheme were composed of two major steps: first, tumor segmentation was conducted manually; and thereafter, texture features were calculated automatically. The manual segmentation of neoplasms in the anterior mediastinum was performed independently by a chest radiologist. The region of interest (ROI) was depicted around the border of each tumor (**Figure 3**). After segmenting a 3-dimensional volume of interest (3D-VOI), texture features were automatically calculated and extracted. In addition, another chest radiologist segmented 30 cases including 15 pathologically proven TETs and 15 lymphomas randomly selected from all samples to evaluate the inter-operator variability. Features with intraclass correlation coefficient value higher than 0.8 were considered stable and used for model construction. The definition included the following criteria : 1) calcification, hemorrhage, liquefaction, necrosis, and blood vessels within the lesion with the largest diameter <2 mm in the tumor were regarded as components of the lesion, and will be included ; 2) the soft-tissue boundary avoided surrounding fat, metal stents, blood, and other structures ; 3) to eliminate partial volume effect, the outline boundary was ≤1 mm of the lesion, and the first and last layers of the lesion were removed.

Before performing features calculations, “Radiomics” automatically resampled the 3D-VOI to a pixel pitch of 1.0 mm in three anatomical directions to reduce the impact of pixel size and thickness. In the original VOIs, different filters would be applied, such as the Laplacian of Gaussian filtering, wavelet filtering, nonlinear intensity transformation, and others. Finally, 1226 radiomics features were as follows : 1) 17 shape and size features, 2) 18 first-order features, 3) 65 texture features, 4) 1116 high-order features and features that have undergone multiple mathematical transformations (wavelet, square, square root, logarithm, and exponents decompositions of first-order statistics and texture features), were extracted from the original and post-processed 3D-VOI of every patient.

### Feature Selection and Model Building

All patients were first splitted into training cohort and test cohort with a ratio of 7:3 by using the stratified random sampling technique: 171 patients (mean age, 43.2 ± 14.8 years; TETs, 105 cases; lymphoma, 66 cases) were allocated into the training cohort; 71 patients (mean age, 42.7 ± 15.7 years; TETs, 44 cases; lymphoma, 27 cases) were allocated into the test cohort. Training cohort was used to train the model based on the least absolute shrinkage and selection operator regression model with tuned parameter of lambda. Since there was a class imbalance that might affect the model tuning, synthetic minority over-sampling technique was applied to the training cohorts before formal model training. The lambda was tuned across multiple values between 0.01-0.2 and the optimum value was decided when the model gained the highest AUC by 5 repeats 10-fold cross-validation. In addition, this performance of optimal setting was also recorded. Next, the feature importance ranking list was derived based on this least absolute shrinkage and selection operator model. Appropriate number of features were selected on this order of importance to simplify the radiomics multivariate logistic regression model, based on acceptable performance relative to the optimal setting.

Regarding the clinical and radiologic features, univariate logistic regression was applied. In addition, features with statistical significance (*P*<0.05) were selected. The features enrolled in the radiomics and clinico-radiologic model were the candidates for the combined model. Similarly, those features with no statistical significance (*P*≥0.05) in the combined model were removed to ease the redundancy of the model.

The performance of the model according to receiver operator curves (ROC) was evaluated in training and independent test cohorts, respectively. Besides the diagnostic performance-related statistics by these two cohorts regarding sensitivity, specificity, and accuracy were illustrated using a confusion matrix. Finally, decision curve analysis was used to evaluate clinical utility of the three models in the training cohort. The workflow of this study is shown in **Figure 3**.

### Statistical Analysis

All statistical analyses were performed using R software (version 1.1.453) and SPSS (version 26, SPSS Chicago, IL). Qualitative variables were presented as frequencies. Normally distributed variables were shown as the mean ± SD (standard deviation). Between TETs and lymphomas (two groups), the clinico-radiologic characteristics were compared by using the chi-square test or Fisher’s exact test for categorical variables and independent t-test or the Mann-Whitney U test for continuous variables. *P*<0.05 indicated statistical difference. Univariate logistic regression analysis was performed for those parameters that showed *P*<0.10 when compared between two groups. The results of univariate logistic regression analysis are shown as OR (95% CI) and *P* value in **Tables 1** and **2**. Inter-operator variability of the radiomics features was assessed with ICC. Interclass correlation coefficient (≦0.40, poor agreement; 0.41–0.60, moderate agreement; 0.61–0.80, good agreement; and>0.80, excellent agreement). The ROCs of the radiomics model in the two cohorts were compared with the DeLong test to evaluate whether overfitting occurred. Decision curve analysis was performed to determine the clinical usefulness of the three models by calculating the net benefits at different threshold probabilities in the whole cohort. The net benefit is equivalent to the proportion of net true positives in brief.

## Results

### Basic Clinico-Radiologic Characteristics

Of the 242 patients, 149 patients were diagnosed with TETs and 93 patients with lymphomas. Compared with patients with lymphoma, patients with TETs had a later age of onset (*P*<0.001) and showed less chest pain (*P*=0.004), respiratory symptoms (*P*<0.001), and lymphadenopathy (*P*<0.001). Regarding gender distribution and B symptoms, no significant differences were found between the two groups (*P*=0.600, 0.200, respectively). In laboratory tests, compared with lymphoma patients, TETs patients had higher lymphocyte counts (*P*<0.001), lower platelet counts (*P*<0.001), and lower lactate dehydrogenase (*P*<0.001), while red blood cell counts and white cell counts showed no significant difference (*P*=0.700, *P*=0.056, respectively). Details of the demographical data and clinical characteristics of the training and test cohorts are summarized in **Table 2**.

For comparison of the radiologic features in TETs and lymphoma, there was no significant difference about location distribution between the two groups (*P*=0.200). The TETs group had a smaller tumor diameter than the lymphoma group (*P*<0.001), but higher CT value and NEV (*P*<0.001, *P*=0.001, respectively). Compared with the lymphoma group, more obvious fat gap between the lesion and big vessels (pulmonary trunk and ascending aorta), less pleural and pericardial effusion existed in the TETs group (all *P*<0.001). Less necrosis, better density uniformity, and clearer boundary of lesions were found in the TETs group (all *P*<0.001) than the lymphoma group. More comprehensive information is listed in **Table 1**.

### Model Building

**Figure 4:**
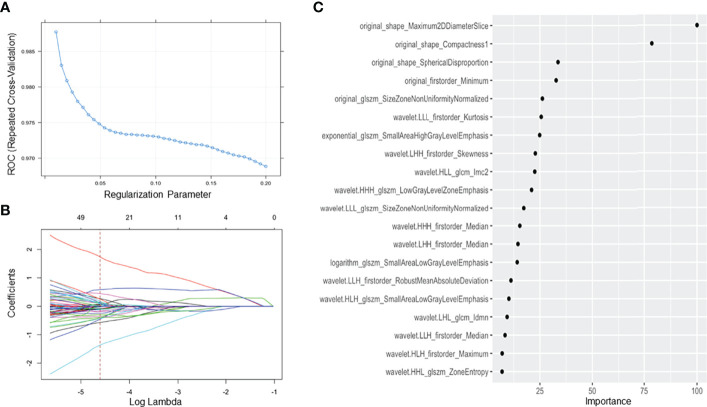
Feature selection using the least absolute shrinkage and selection operator regression method. **(A)** The tuned parameter (λ) in the LASSO model was selected *via* 5 repeats 10-fold cross-validation based on minimum criteria. The dotted blue curve indicates the average binominal deviance values for each model with a given λ. The λ value was set as 0.01 in this study; **(B)** the dotted vertical line was plotted at the selected λ value, resulting in 20 radiomics features; **(C)** sorting the importance of radiomics features, and the top 5 features were included in our model.

Among 242 patients in our study, 171 patients were allocated into the training cohort, while 71 patients were in the test cohort. A total of 385 radiomics features were shown to be stable (good and excellent agreement), including 17 shape and size features, 13 first-order features, 34 texture features, and 321 high-order features and features that have undergone multiple mathematical transformations. After SMOTE, the training cohort was up-sampled to the number of 462 with a class ratio of 264:198 for TETs:lymphoma. An AUC of 0.981 (95% CI: 0.971–0.991) suggested that there was no significant affect toward the model tuning from the imbalance between the patient groups in our study. After the tuning process, the optima Lambda was set to be 0.01 for our dataset. The mean AUC for each lambda tuned with our resample by 5-repeated 10-fold cross-validation is shown in **Figure 4A**. In addition, the optimal AUC was reached beyond 0.9. For each tuned lambda, the coefficients of the features are shown in **Figure 4B**. Moreover, the importance ranking list based on the model is shown in **Figure 4C**.

In our study, the regression model with the top 5 features on the order of importance gave us acceptance AUC relative to the optimal setting. Moreover, these five radiomics features by 3D texture analysis in original images included three shape-related features, one first order related feature, and one feature about gray level size zone matrix (GLSZM). Three shape-related features included maximum 2D diameter slice, compactness 1, and spherical disproportion. Minimum as a first-order feature represented minimum eigenvalue and size zone nonuniformity normalized was a feature calculated from GLSZM. Three clinico-radiologic characteristics, including lymphadenopathy (+/-), myasthenia gravis (+/-), and pericardial effusion (+/-), constituted the clinico-radiologic model. For the final combined model, all the features in the radiomics and clinico-radiologic models were enrolled except lymphadenopathy (+/-) which was removed as redundant.

### Model Validation and Comparison

**Figure 5:**
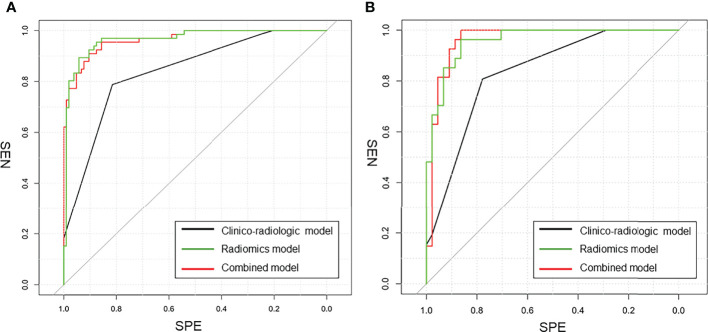
The comparison of ROC curves in this study. **(A)** in the training cohort. AUC = 0.975 for the combined model, 0.860 for the clinico-radiologic model, and 0.965 for the radiomics model; **(B)** in the external validation cohort. AUC = 0.955 for the combined model, 0.843 for the clinico-radiologic model, and 0.961 for the radiomics model.

The ROC results of three models are shown in **Table 4** and **Figure 5**. The performance of the radiomics model was good in the training group with an AUC of 0.965 (95% CI: 0.941-0.990). The classification accuracy, sensitivity, and specificity were 89.5%, 83.3%, and 93.3%, respectively. Good performance was also observed in the test group with AUC being 0.961 (95% CI: 0.917–1.00). The accuracy, sensitivity, and specificity were 90.1%, 92.6%, and 88.6%, respectively. For the clinico-radiologic model, all performance metrics were lower than that of the radiomics model excluding specificity. The AUCs were 0.860 (95% CI: 0.808–0.913) and 0.843 (95% CI: 0.759–0.928) in the training group and test group, respectively. The classification accuracies were 83.6% and 84.5% in the 2 groups, respectively.

**Table 4:** Differentiation performance of clinico-radiologic model, radiomics model, and combined model in the training and test cohorts.

Variables	Training cohort				Test cohort			
	AUC	ACC (%)	SEN (%)	SPE (%)	AUC	ACC (%)	SEN (%)	SPE (%)
Clinico-radiologic model	0.860 (95% CI: 0.808-0.913)	83.6	71.2	91.4	0.843 (95% CI: 0.759-0.923)	84.5	70.4	93.2
Radiomics model	0.965 (95% CI: 0.941-0.990)	89.5	83.3	93.3	0.961(95% CI: 0.917-1.000)	90.1	92.6	88.6
Combined model	0.975 (95% CI: 0.956-0.995)	91.8	89.4	93.3	0.955(95% CI: 0.915-0.996)	85.9	81.5	88.6

AUC, area under the summary receiver operating characteristic curve; ACC, accuracy; SEN, sensitivity; SPE, specificity; CI, confidence interval.

In the combined model, AUCs in the training group and independent test group were 0.975 (95% CI: 0.956-0.995) and 0.955 (95% CI: 0.915-0.996), respectively; while the classification accuracies were 89.5% and 90.1%, respectively. In addition, the combined model showed comparative diagnostic performance to radiomics signature model. The performance of the radiomics model and the combined model were both significantly higher than that of the clinical model.

**Figure 6:**
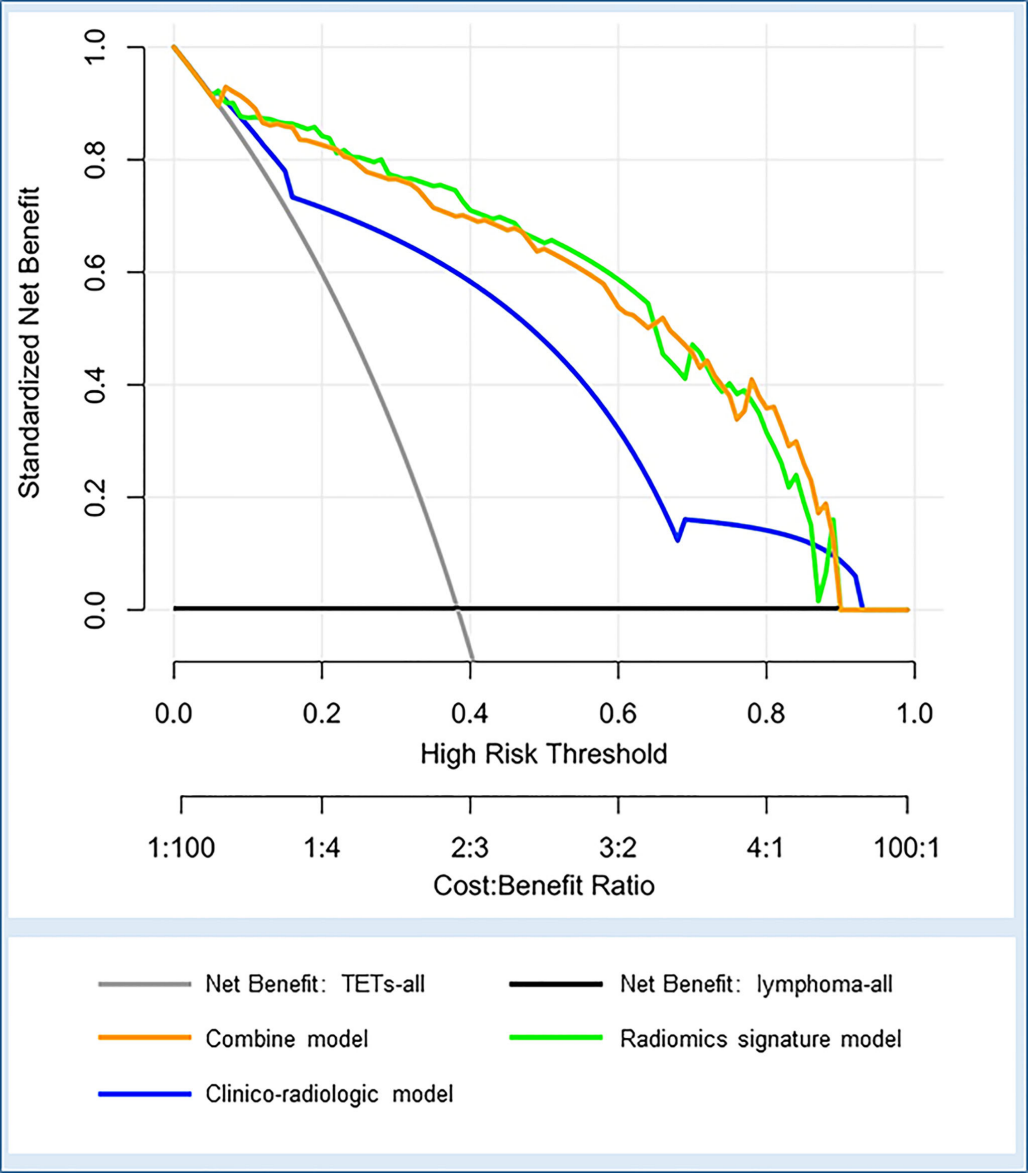
Decision curve analysis for the three models in the whole cohort. The net benefit vs. the threshold probability is plotted. The x-axis shows the threshold probability. The y-axis shows the net benefit. A model is only clinically useful if it has a higher net benefit than the default diagnosis of TETs-all and lymphoma-all. The two curves (orange and green curves) indicate that the combined model and the radiomics signature model are superior to the diagnosis of TETs-all (gray line), the diagnosis of lymphoma-all (black line), and the clinico-radiologic model (blue curve) within a threshold probability of 5%-90% (orange curve) and 10%-87% (green curve).

Furthermore, decision curve analysis demonstrated that the combined model and radiomics signature model would offer net benefits over the “TETs-all”, “lymphoma-all”, and the clinico-radiologic model within a certain range of threshold (5%–90% for combined model; 10%–87% for radiomics signature model) in the training cohort (**Figure 6**). Moreover, the combined model and the radiomics signature model showed comparable net benefits.

## Discussion

Due to the differences in therapeutic approaches, accurate diagnosis of TETs and lymphomas before treatment is of great significance for clinical decision (6, 18). CT is a routine examination for chest diseases including mediastinal lesions and lung diseases. Nevertheless, imaging methods such as CT and magnetic resonance imaging are still challenging in accurate diagnosis of mediastinal masses in clinical practice. Tomiyama et al. (19) reported that for neoplasms in anterior mediastinum, the diagnostic accuracies of CT and MRI for thymoma, thymic carcinoma, and lymphoma were 83%, 13%, 55% and 84%, 13%, 43%, respectively. In the study of Ackman et al. (4), unnecessary or non-therapeutic thymectomy accounted for 43.8% (70/160) of the 160 thymectomy cases including lymphomas (54.3%, 38/70), thymic bed cysts (24.3%, 17/70), and other lesions. The unclear preoperative diagnosis in some patients has led to many unnecessary surgical treatments. Recently, newly emerged radiomics showed potential in accurate differentiation of different space-occupying lesions before treatment using the texture feature analysis. Kayi Cangir et al. (20) revealed that radiomic signature using the k-nearest neighbor classier based on enhanced CT had excellent efficacy for discriminating low- vs. high-risk thymoma groups with an AUC of 0.943 in the validation cohort. As CT is more widely used and it serves as the standard-of-care imaging tool for pre-treatment evaluation of anterior mediastinal diseases, it is more convenient and effective to construct radiomics models using features extracted from routinely obtained contrast CT images. In our study, we developed and validated CT-based radiomics models for non-invasive differentiation of TETs and lymphomas. Our results showed that the AUCs of the radiomic model based on enhanced CT in the training and test cohorts for TETs and lymphomas differentiation were outstanding (0.965 and 0.961, respectively). In addition, this model, with the accuracies of 89.5% and 90.1% in training and test cohorts, respectively, is superior to traditional CT in distinguishing the two sorts of tumors. A study by Kirienko et al. (16) described that a radiomic model to differentiate TETs and lymphomas based on non-enhanced chest CT showed AUCs of 0.93 and 0.84 in the training and test cohorts, respectively. The accuracies of their model in the training cohort and test cohort were 91.3% and 76.9%, respectively, and it was also better than conventional CT in distinguishing TETs and lymphomas. Compared with the study by Kirienko et al. (16), our study had better results with higher AUCs in two cohorts and better stability between the training cohort and the test cohort, which may be explained by that our study had a larger sample size and the radiomics features were extracted from contrast-enhanced CT images. It is worth noting that a significant enhancement difference between TETs and lymphomas was found in our study, which is consistent with the results of some previous studies (21, 22). Significant blood supply differences exist between the two sorts of tumors. At the fine scale, texture features extracted from the enhanced CT images might represent the distribution of contrast media in the extracellular space between intra- and extravascular (23). Thus, radiomics signature from enhanced CT may be more effective in showing the internal heterogeneity between TETs and lymphoma. In addition, in the study by Huet et al. (24), for the differentiation of high-risk TETs and low-risk TETs, the enhanced CT-based radiomics model using the random forest machine learning classifier achieved an AUC of 0.81, which was better than the AUC of 0.61 by non-enhanced CT-based radiomics model. For tumors at other sites, radiomics from enhanced CT showed excellent performances in assessment of colorectal cancer heterogeneity and differentiation of benign and malignant gallbladder polypoid lesions (22, 24).

Furthermore, based on the obvious enhancement difference between TETs and lymphomas, different techniques were also used to explore the differential value between the two types of tumors. In previous studies, triple-phase CT spectral imaging and contrast-enhanced ultrasound imaging had been used in differentiating thymic neoplasms and lymphomas with the best AUCs of 0.875 and 0.668, respectively (21, 22). In the study by Sakai et al. (25),the accuracy for differentiating thymoma and non-thymoma by dynamic magnetic resonance imaging was 81%. In our study, the radiomics model based on enhanced CT with a best AUC of 0.961 and accuracy of 90.1% had a better differentiation efficacy than the studies above. Compared with imaging features on spectral CT, contrast-enhanced ultrasound, and dynamic magnetic resonance imaging, radiomics signature from contrast enhanced CT also showed superior differential performance between TETs and lymphoma (21, 22, 25). In addition, ^18^F-FDG PET-CT and whole-body MRI could assess the patients’ general condition, which were meaningful to anterior mediastinal primary lymphomas with multiple systemic involvement. It is well known that PET-CT is used for pre-treatment staging, treatment efficacy evaluation, and post-treatment follow-up in patients with lymphoma (26, 27). Further, Lei et al. (28) explored the performance of metabolism parameters, including SUVmean, SUVmax, TLG, and MTV, of ^18^F-FDG PET-CT for distinguishing TETs from lymphomas. The performance of the study of Lei et al. (28) with best AUC of 0.767 and best accuracy of 72.8% in SUVmax and SUVmin, respectively, was inferior to ours. In general, lymphomas have higher FDG uptake than TETs (28). However, the lower metabolic activity of indolent lymphomas and the markedly different metabolic activity associated with the grade of TETs may be accountable for the unsatisfactory results of metabolic parameters for the differentiation of the two kinds of tumors (27, 29–31). Regrettably, there was no whole-body MRI study in this topic. In addition, whole-body MRI with advantages in whole-body scanning may perform better than chest MRI. Radiomics signature may be related to some of the biological behavior of the tumors as it is able to mine more image information invisible to the naked eyes and objectively quantify the features. These features may be well associated with heterogeneity of the lesion itself (32, 33). Additionally, no extra-cost is needed for the patients.

In terms of clinical factors, lymphadenopathy (–) and myasthenia gravis (+) included in our model revealed a higher risk of patients with TETs than with lymphoma, which was consistent with previous research (16). The top three clinico-radiologic features in the ranking list based on this model with optimal lambda contained myasthenia gravis rather than autoimmune disease. For malignant tumors, pericardial effusion can be caused by pericardial involvement even in patients without symptoms or with atypical symptoms (34). The most common causes of pericardial effusion caused by tumor involvement were lung cancer, breast cancer, and hematologic tumors such as leukemia, Hodgkin’s lymphoma, and non-Hodgkin’s lymphoma (35). However, pericardial effusion occurred when thymic malignancies invaded the pericardium, but this was uncommon, even if the primary lesion was often in the anterior mediastinum (23). In our study, lymphomas were mainly non-inert growth types as shown in **Figure 2**. In addition, the larger the lymphoma was, the more likely concomitant of local and systemic symptoms such as chest pain, respiratory symptoms, and B symptoms, which may be related to the lymphoma’s greater growth capacity and aggressiveness to surrounding tissues (**Table 2**).

In addition, we further established a combined model based on radiomics features and clinico-radiologic characteristics. Compared with the radiomics signature model, the combined model did not show a significant improvement in discriminative efficacy, which yielded AUCs of 0.975 and 0.955, and accuracies of 91.8% and 85.9% in the training and test cohorts, respectively. The superior performance of the combined model in this study may be attributed to the inclusion of radiomics signature, which contain many quantitative features, especially parameters not easy to be obtained through simple visual analysis or conventional imaging tools. Both the radiomics features model and the combined model in the current study had outstanding and comparable performance in distinguishing TETs and lymphomas and can provide a net benefit superior to the diagnosis of “TETs-all”, the diagnosis of “lymphoma-all”, and the clinico-radiologic model in the decision curve.

There were several limitations in our study. First, selection bias maybe came from the research nature, a retrospective study from a single center. Second, this study included patients over a longer period; thus, the images were collected from CT scanners of different vendors; however, a good result proved the generalization ability of this radiomics signature model. Finally, the imbalance between groups may affect model tuning. However, the synthetic minority over-sampling technique used in the training group found that the ROC of the model was 0.981 (95% CI: 0.971–0.991) and the imbalance between groups did not affect the model performance.

## Conclusion

The radiomics signature model and combined model exhibit outstanding and comparable differential diagnostic performances between TETs and lymphomas. The CT-based radiomics analysis might serve as an effective tool for accurate differentiating TETs from lymphomas before treatment in clinical practice.

## Data Availability Statement

The original contributions presented in the study are included in the article/supplementary material. Further inquiries can be directed to the corresponding author.

## Author Contributions

WH, CX, and LP: study design. WH and CX: data collection. WH and CX: data processing. All authors: manuscript writing. All authors: manuscript revision. All authors: final approval of manuscript. All authors contributed to the article and approved the submitted version.

## Funding

This work was supported by the National Natural Science Foundation of China (No. 81601462) and the Key Research & Development Project of Science and Technology of Sichuan Province (No. 2021YFS0142).

## Conflict of Interest

Author XinC was employed by the company Siemens Healthineers.

The remaining authors declare that the research was conducted in the absence of any commercial or financial relationships that could be construed as a potential conflict of interest.

## Publisher’s Note

All claims expressed in this article are solely those of the authors and do not necessarily represent those of their affiliated organizations, or those of the publisher, the editors and the reviewers. Any product that may be evaluated in this article, or claim that may be made by its manufacturer, is not guaranteed or endorsed by the publisher.
